# Supplementary material for: Functional dichotomy and distinct nanoscale assemblies of a cell cycle-controlled bipolar zinc-finger regulator
Source: eLife. 2016 Dec 23;5:e18647. doi: 10.7554/eLife.18647 (PMC5182063; doi:10.7554/eLife.18647)
Supplement: Supplementary file 1. — DOI: http://dx.doi.org/10.7554/eLife.18647.017 [file elife-18647-supp1.doc]

**SUPPLEMENTARY INFORMATION**

## Strains

|  | **Characteristics** | | | **Reference/source** |
| --- | --- | --- | --- | --- |
| ***Escherichia coli*** | |  | |  |
| S17-1 | RP4,Tc::Mu Km::Tn7 | | | (Simon et al., 1983) |
| EC100D | *F- mcrA Δ(mrr-hsdRMS-mcrBC) Φ80dlacZΔM15 ΔlacX74 recA1 endA1 araD139 Δ(ara, leu)7697 galU galK λ- rpsL (StrR) nupG* | | | Epicentre |
| Rosetta (DE3) | F^-^ *ompT hsdS*_B_(r_B_^-^ m_B_^-^) *gal dcm* (DE3) pLysSRARE (Cam^R^) | | | EMD Millipore, Billerica, MA |
| XL1-Red | *endA1 gyrA96 thi-1 hsdR17 supE44 relA1 lac mutD5 mutS mutT* Tn10 (Tet^R^) | | | Agilent Technologies |
|  | | |  |  |
| ***Caulobacter crescentus*** | | |  |  |
| NA1000 | Synchronizable derivative of wild-type strain CB15 | | | (Evinger and Agabian, 1977) |
| LS3118 | NA1000 Δ*pilA* | | | (Skerker and Shapiro, 2000a) |
| LS3216 | NA1000 Δ*cpaC* | | | (Skerker and Shapiro, 2000a) |
| LS3218 | NA1000 Δ*cpaE* | | | (Skerker and Shapiro, 2000a) |
| PV28 | NA1000 Δ*pleA* | | | (Viollier and Shapiro, 2003) |
| PV14 | NA1000 ΔpilA-cpaF::Ωaac3 | | | (Viollier et al., 2002b) |
| PV17 | NA1000 Δ*podJ* | | | (Viollier et al., 2002b) |
| UJ506 | NA1000 Δ*pleC* | | | (Aldridge et al., 2003) |
| TPA2357 | NA1000 Δ*fljx6 (ΔflkJKLMNO)* | | | (Faulds-Pain et al., 2011) |
| LS3196 | NA1000 Δ*divJ*::*gent^R^* | | | (Wheeler and Shapiro, 1999) |
| LS3570 | NA1000 *divK^cs^* | | | (Hung and Shapiro, 2002) |
| GB255 | NA1000 Δ*popZ*::Ω | | | (Bowman et al., 2008) |
| LT407 | NA1000 Δ*gcrA*::Ω | | | (Murray et al., 2014) |
| LS3707 | NA1000 Δ*gcrA*::Ω *xylX::*P*_xyl_-gcrA* | | | (Holtzendorff et al., 2004) |
| UG2215 | NA1000 Δ*ccrM*::Ω | | | (Fioravanti et al., 2013) |
| PV418 | NA1000 *yfp-cpaE* | | | (Viollier et al., 2002a) |
| FNE101 | NA1000 *zitP::himar1* (nt 2445704) | | | This work |
| FNE124 | NA1000 *zitP::himar1* (nt 2445734) | | | This work |
| FNE158 | NA1000 *zitP::himar1* (nt 2445308) | | | This work |
| FBE10 | NA1000 *zitP::himar1* (nt 2445309) | | | This work |
| FNE106 | NA1000 *cpaM::himar1* (nt 3708907) | | | This work |
| FNE126 | NA1000 *cpaM::himar1* (nt 3709648) | | | This work |
| FNP5 | NA1000 *cpaM::himar1* (nt 3709444) | | | This work |
| UG4505 (JM1) | NA1000 Δ*zitP* | | | This work |
| UG4506 (JM2) | NA1000 Δ*cpaM* | | | This work |
| JM3 | NA1000 *xylX::*P*_xyl_-dendra2-zitP* | | | This work |
| JM4 | NA1000 Δ*zitP xylX::*P*_xyl_-dendra2-zitP* | | | This work |
| JM5 | NA1000 Δ*zitP xylX::*P*_xyl_-dendra2-zitP^CS^* | | | This work |
| JM6 | NA1000 Δ*zitP xylX::*P*_xyl_-dendra2-zitP^GAP^* | | | This work |
| JM7 | NA1000 Δ*cpaM xylX::*P*_xyl_-dendra2-zitP* | | | This work |
| JM8 | NA1000 Δ*divJ xylX::*P*_xyl_-dendra2-zitP* | | | This work |
| JM9 | NA1000 Δ*pleC xylX::*P*_xyl_-dendra2-zitP* | | | This work |
| JM10 | NA1000 Δ*popZ::Ω xylX::*P*_xyl_-dendra2-zitP* | | | This work |
| JM11 | NA1000 *divK^cs^ xylX::*P*_xyl_-dendra2-zitP* | | | This work |
| JM12 | NA1000 *xylX::*P*_xyl_-dendra2-cpaM* | | | This work |
| JM13 | NA1000 Δ*cpaM xylX::*P*_xyl_-dendra2-cpaM* | | | This work |
| JM14 | NA1000 Δ*cpaM xylX::*P*_xyl_-dendra2-cpaM^MAS^* | | | This work |
| JM15 | NA1000 Δ*zitP xylX::*P*_xyl_-dendra2-cpaM* | | | This work |
| JM16 | NA1000 Δ*divJ xylX::*P*_xyl_-dendra2-cpaM* | | | This work |
| JM17 | NA1000 Δ*pleC xylX::*P*_xyl_-dendra2-cpaM* | | | This work |
| JM18 | NA1000 Δ*popZ::Ω xylX::*P*_xyl_-dendra2-cpaM* | | | This work |
| JM19 | NA1000 *divK^cs^ xylX::*P*_xyl_-dendra2-cpaM* | | | This work |
| JM20 | NA1000 Δ*zitP yfp-cpaE* | | | This work |
| JM21 | NA1000 Δ*cpaM yfp-cpaE* | | | This work |
| JM22 | NA1000 *fliG^D306G^* | | | This work |
| JM23 | NA1000 Δ*zitP;fliG^D306G^* | | | This work |
| MB1250 | NA1000 Δ*zitP xylX::*P*_xyl_-dendra2-zitP^1-133^* | | | (Bergé et al., in revision) |

## Plasmids

|  | **Characteristics** | **Reference/source** |
| --- | --- | --- |
| pHPV414 | non replicative vector in *C. crescentus* harboring the *himar1* transposon (Kan^R^) | (Viollier et al., 2004) |
| pNPTS138 | non replicative vector in *C. crescentus* containing the *sacB* gene (Kan^R^) | M.R.K. Alley, unpublished |
| pNPTS138-hook | pNPTS138 allowing the backcross of *fliG^D306G^* allele (double recombination, sucrose counter selection) | (Fumeaux et al., 2014) |
| pNPTS138-Δ*zitP* | pNPTS138 allowing *zitP* deletion (double recombination, sucrose counter selection) | This work |
| pNPTS138-Δ*cpaM* | pNPTS138 allowing *cpaM* deletion (double recombination, sucrose counter selection) | This work |
| pET28a | High copy number vector for expression of genes under the control of T7 promoter | Novagen, Madison, WI |
| pET28a-*zitP^Cterm^* (pCWR334) | pET28a containing the *zitP^Cterm^* truncated allele | This work |
| pET28a-*cpaM^ΔTM^* | pET28a containing the *cpaM^ΔTM^* truncated allele | This work |
| pMT335 | High copy number vector containing the leaky P*_van_* inducible promoter (Gent^R^) | (Thanbichler et al., 2007) |
| pCWR512 | Low copy number vector containing *pflI*-TAP fusion gene under the control of the leaky P*_van_* inducible promoter (Gent^R^) | (Davis et al., 2013) |
| pCWR512-P*_zitP_*-*zitP* | pCWR512 containing *zitP*-TAP fusion gene in place of *pflI*-TAP under the control of native *zitP* promoter | This work |
| pMT335*-zitP* | pMT335 containing *zitP* coding sequence | This work |
| pMT335*-zitP^CS^* | pMT335 containing the *zitP^CS^* mutated tetracysteine allele | This work |
| pMT335*-zitP^CA^* | pMT335 containing the *zitP^CA^* mutated tetracysteine allele | This work |
| pMT335*-zitP^GAP^* | pMT335 containing the *zitP^GAP^* mutant allele | This work |
| pMT335-P*_zitP_*-*zitP* | pMT335 containing *zitP* promoter and coding sequence | This work |
| pMT335-P*_zitP_*-*zitP^CS^* | pMT335 containing the *zitP^CS^* mutated tetracysteine allele under native *zitP* promoter control | This work |
| pMT335-P*_zitP_*-*zitP^CA^* | pMT335 containing the *zitP^CA^* mutated tetracysteine allele under native *zitP* promoter control | This work |
| pUG78 | pMT335 containing *cpaM* coding sequence | This work |
| pMT582 | Kan^R^ integrative plasmid at *C. crescentus* *xylX* locus allowing xylose inducible GFP N-terminal fusions | (Thanbichler et al., 2007) |
| pX-*ftsZ*-*dendra2* | Kan^R^ integrative plasmid at *C. crescentus* *xylX* locus allowing xylose inducible production of the FtsZ-Dendra2 C-terminal fusions | (Biteen et al., 2012) |
| pX-*dendra2*N2 | pMT582 derivative allowing xylose inducible Dendra2 N-terminal fusions. | This work |
| pX-*dendra2*N2-*zitP* | pX-*dendra2*N2 containing *zitP* coding sequence | This work |
| pX-*dendra2*N2-*zitP^CS^* | pX-*dendra2*N2 containing the *zitP^CS^* mutated tetracysteine allele | This work |
| pX-*dendra2*N2-*zitP****^GAP^*** | pX-*dendra2*N2 containing the *zitP^GAP^* mutated allele | This work |
| pX-*dendra2*N2-*zitP^1-133^* | pX-*dendra2*N2 containing the *zitP^1-133^* allele | (Bergé et al., in revision) |
| pX-*dendra2*N2-*cpaM* | pX-*dendra2*N2 containing *cpaM* coding sequence | This work |
| pJS70 | p*lacZ*290 containing *pilA* promoter region | (Skerker and Shapiro, 2000b) |
| pP-*CC_0095* | p*lacZ*290 containing *CC_0095* promoter region | (Fumeaux et al., 2014) |
| pP-*CC_1982* | p*lacZ*290 containing *CC_1982* promoter region | (Fumeaux et al., 2014) |
| P*_tipF_*-*lacZ* | p*lacZ*290 containing *tipF* promoter region | (Fioravanti et al., 2013) |
| p*lacZ*290-p*ftsN* | p*lacZ*290 containing *ftsN* promoter region | (Murray et al., 2014) |
| P*_podJ_*-*lacZ* | p*lacZ*290 containing *podJ* promoter region | (Fioravanti et al., 2013) |
| pP-*ctrA* | p*lacZ*290 containing *ctrA* promoter region | (Fumeaux et al., 2014) |
| p*fljL*/*lacZ*/290 | p*lacZ*290 containing *fljL* promoter region | (Mangan et al., 1995) |
| P*_pstC_*-*lacZ* | p*lacZ*290 containing *pstC* promoter region | (Lubin et al., 2015) |
| *plac290*-*P_spmX_-lacZ* | p*lacZ*290 containing *spmX* promoter region | (Radhakrishnan et al., 2008) |
| pP*_higBA_-lac*290 | p*lacZ*290 containing *higB* promoter region | (Kirkpatrick et al., 2016) |
| pMT463 | High copy plasmid carrying a Pxyl promoter (gentR) | (Thanbichler et al., 2007) |
| pMT463-*zitP* | pMT463 containing *zitP* coding sequence | This work |
| pMT463-*zitP^1-133^* | pMT463 containing *zitP^1-133^* allele | This work |
| pXTCYC-4 | Integrative plasmid at *C. crescentus* *xylX* locus allowing xylose inducible | (Thanbichler et al., 2007) |
| pXTCYC-4-*relA′*-FLAG | *relA′*-FLAG under the control of the *xylX* promoter in pXTCYC-4 | (Gonzalez and Collier, 2014) |

## Oligonucleotides

| **Names** | **Sequences** |  |
| --- | --- | --- |
| 2215_1_Eco | 5’-AAAAAAGAATTCGGCTTCCAGCGAGAGACCTGAAAGA-3’ |  |
| 2215_2_bam bis | 5’-AAAAAAGGATCCATAGCGGCTGGCGCACTCCGGGCA-3’ |  |
| 2215_3_bam | 5’-AAAAAAGGATCCTCTCACGAACCCGCTCATCATGAA-3’ |  |
| 2215_4_hind | 5’-AAAAAAAAGCTTCGGCCATGGCCAGCGAGTACAATA-3’ |  |
| 3440_1_Eco | 5’-AAAAAAGAATTCGCGTCACAAGGGGACAAATT-3’ |  |
| 3440_2_bam | 5’-AAAAAAGGATCCCGAAAACGAGACGGCCATGA-3’ |  |
| 3440_3_bam | 5’-AAAAAAGGATCCTCGGCCCTGGCGCATCGCT-3’ |  |
| 3440_4_hind | 5’-AAAAAAAAGCTTCCCGCACAGCCATAGCCAT-3’ |  |
| 2215CTD-NdeI | 5’-AAAAAACATATGGAGCTGCTGAGCAAGGAAGAAAA-3’ |  |
| 2215CTD-EcoRI | 5’-AAAAAAGAATTCATTCATGATGAGCGGGTTCGTGAGA-3’ |  |
| 2215_CTF_RI | 5’-AAAAAAGAATTCTTCATGATGAGCGGGTTCGT-3’ |  |
| 3440_nde_His | 5’-AAAAAACATATGGTGATTCGCCTCGAGCTGA-3’ |  |
| 3440_Eco | 5’-AAAAAAGAATTCAGCGATGCGCCAGGGCCGA-3’ |  |
| CC2215_n | 5’-aaaaaacatatgtcgatccgcaaggcgcgtca-3’ |  |
| 2215sh_NdeI | 5’-AAAAAACATATGATACTGACCTGCCCGGA-3’ |  |
| CC2215_E | 5’-AAAAAAGAATTCATTCATGATGAGCGGGTTCGTGAGA-3’ |  |
| 2215shCS_NdeI | 5’-AAAAAACATATGATACTGACCTCCCCGGA-3’ |  |
| 2215shCA_NdeI | 5’-AAAAAACATATGATACTGACTGCCCCGGA-3’ |  |
| 3440_nde | 5’-AAAAAACATATGGCCGTCTCGTTTTCGCGCAA-3’ |  |
| dendra2N_F | 5’-ACGACCATATGAACACCCCGGGAATTAACC-3’ |  |
| dendra2N_R | 5’-GCATATTAATTAAGGCGCCTGCAGGCCACACCTGGCTGGGCAGG-3’ |  |
| 2215sh+2_SacI | 5’-AAAAAAGAGCTCTAATGATACTGACCTGCCCGGA-3’ |  |
| 2215shCS+2_SacI | 5’-AAAAAAGAGCTCTAATGATACTGACCTCCCCGGA-3’ |  |
| 3440_+2_SacI | 5’-AAAAAAGAGCTCTAATGGCCGTCTCGTTTTCG-3’ |  |
| DUF3426_1_Eco | 5’-AAAAAAgaattcAGCAGCCGGAACTCCTGGAAGACCA-3’ |  |
| DUF3426_2_bam | 5’-AAAAAAGGATCCGATGCTGCCGGCGTCGATCACCA-3’ |  |
| DUF3426_3_bam | 5’-AAAAAAgaattcCAGGAACCCCCGGGACATGAGA-3’ |  |
| DUF3426_4_Hind | 5’-AAAAAAAAGCTTGGCGCGGCCATGGCCAGCGAGTACA-3’ |  |
| 2215_DUF_RI | 5’- AAAAAAGAATTCTCAGACATCGATCCGGAAGAT-3’ |  |

*Plasmid constructions*

All DNA fragments were amplified by PCR using the Phusion high fidelity polymerase (www.thermoscientificbio.com/) following a protocol as recommended by the manufacturer. PCR products were then transferred in dedicated expression vectors and sequence-verified. All genome coordinates refer to the NA1000 geneome.

pNPTS138-Δ*zitP*. The plasmid construct used to delete *zitP* (*ccna_02298* or *cc_2215*) from NA1000 creating strain UG4505. The deletion construct was made by PCR amplification of two fragments. The first encompasses a 858-bp sequence (nt 2446560-2445703, flanked by an *Eco*RI site at the 5’ end and a *Bam*HI at the 3’ end) was amplified using primers 2215_1_Eco and 2215_2_bam bis. The second encompasses a 826-bp sequence (nt 2444830-2444005, flanked by a *Bam*HI site at the 5’ end and a *Hind*III site at the 3’ end) was amplified using primers 2215_3_bam and 2215_4_hind. The two PCR fragments were first digested with appropriate restriction enzymes and then triple ligated into pNTPS138 (M.R.K. Alley, unpublished) that had been previously restricted with *Hind*III and *Eco*RI. This construct deletes nt 2445702-2444831 or codons 21–310 of the annotated *ccna_02298* coding sequence. (Note that in the NA1000 genome the gene coding sequence for CCNA_02298 is annotated as 319-residue translation product, starting from an upstream GTG rather than the ATG that lies 8 codons downstream would initiate translation of a 311-residue protein).

pNPTS138-Δ*cpaM*. The plasmid construct used for *cpaM* (*ccna_03552* or *cc_3440*) deletion was made by PCR amplification of two fragments. The first encompasses a 657-bp sequence (nt 3708162–3708818 flanked by an *Eco*RI site at the 5’ end and a *Bam*HI at the 3’ end) was amplified using primers 3440_1_Eco and 3440_2_bam. The second encompasses a 606-bp sequence (nt 3709965–3710570, flanked by a *Bam*HI site at the 5’ end and a *Hind*III site at the 3’ end) was amplified using primers 3440_3_bam and 3440_4_hind. The two PCR fragments were first digested with appropriate restriction enzymes and then triple ligated into pNTPS138 (M.R.K. Alley, unpublished) that had been previously restricted with *Hind*III and *Eco*RI. This construct deletes 1146 nt of the *cpaM*-coding sequence (nt 3708819–3709964) or codons 7–388 of the annotated *ccna_03552* coding sequence.

pET28a-*zitP^Cterm^* (pCWR334). The *zitP*-coding sequence from nt 2444803–2445147 encoding the last 114 residues of ZitP was PCR amplified using the 2215CTD-NdeI and 2215CTD-EcoRI primers. This fragment was digested with *Nde*I/*Eco*RI and cloned into *Nde*I/*Eco*RI-digested pET28a (Novagen).

pET28a-*cpaM^ΔTM^*. The *cpaM*-coding sequence lacking the first 189 bp from the start codon (nt 3708996–3709982, encoding the last 329 residues of CpaM) was PCR amplified using the 3440_nde_His and 3440_Eco primers. This fragment was digested with *Nde*I/*Eco*RI and cloned into *Nde*I/*Eco*RI-digested pET28a (Novagen).

pCWR512-P*_zitP_-zitP*. The *zitP*-coding sequence (without stop codon) and promoter (nt 2444806–2445833) were PCR amplified using the CC2215_n and 2215_CTF_RI primers. This fragment was digested with *Nde*I/*Eco*RI and cloned into *Nde*I/*Eco*RI-digested pCWR512 to replace the *pflI* coding sequence (Davis et al., 2013).

pMT335-P*_zitP_-zitP*. The *zitP*-coding sequence and promoter (nt 2444803–2445833) were PCR amplified using the CC2215_n and CC2215_E primers. This fragment was digested with *Nde*I/*Eco*RI and cloned into *Nde*I/*Eco*RI-digested pMT335 (Thanbichler et al., 2007).

pMT335*-zitP*. The *zitP*-coding sequence (nt 2444803–2445738,) was PCR amplified from the NA1000 genome using the 2215sh_NdeI and CC2215_E primers. This fragment was digested with *Nde*I/*Eco*RI and cloned into *Nde*I/*Eco*RI-digested pMT335 (Thanbichler et al., 2007).

pMT335-P*_zitP_-zitP^CS^*. We digested pMT335-P*_zitP_-zitP* and the *2215^CS^* synthetic fragment (dna2.0, Menlo Park, CA, USA) with *Nde*I/*Sac*II and ligated the two fragments.

pMT335-P*_zitP_-zitP^CA^*. We digested pMT335-P*_zitP_-zitP* and the *2215^CA^* synthetic fragment (dna2.0, Menlo Park, CA, USA) with *Nde*I/*Sac*II and ligated the two fragments.

pMT335-*zitP^CS^*. We PCR amplified the *zitP^CS^* tetracysteine mutant allele from pMT335-P*_zitP_-zitP^CS^* with 2215shCS_NdeI and CC2215_E primers. This fragment was digested with *Nde*I/*Eco*RI and cloned into *Nde*I/*Eco*RI-digested pMT335 (Thanbichler et al., 2007).

pMT335-*zitP^CA^*. We PCR amplified the *zitP^CA^* tetracysteine mutant allele from pMT335-P*_zitP_-zitP^CA^* with 2215shCA_NdeI and CC2215_E primers. This fragment was digested with *Nde*I/*Eco*RI and cloned into *Nde*I/*Eco*RI-digested pMT335 (Thanbichler et al., 2007).

pMT335-*zitP^GAP^*. The pMT335-*zitP* was electroporated into the mutagenizing *E coli* XL1-Red strain for random mutagenesis.

pUG78. The *cpaM*-coding sequence (nt 3708801–3709985) was PCR amplified using the 3440_nde and 3440_Eco primers. This fragment was digested with *Nde*I/*Eco*RI and cloned into *Nde*I/*Eco*RI-digested pMT335 (Thanbichler et al., 2007).

pX-*dendra2*N2. We PCR amplified the photoactivatable variant *dendra2* from the pX-*ftsZ*-*dendra2* (Biteen et al., 2012) with dendra2N_F and dendra2N _R primers. This fragment was digested with *Nde*I/*Pac*I and cloned into *Nde*I/*Pac*I-digested pMT582 (Thanbichler et al., 2007).

pX-*dendra2*N2-*zitP*. The *zitP*-coding sequence (nt 2444803–2445738) was PCR amplified using the 2215sh+2_SacI and CC2215_E primers. This fragment was digested with *Sac*I/*Eco*RI and cloned into *Sac*I/*Eco*RI-digested pX-*dendra2*N2.

pX-*dendra2*N2-*zitP^CS^*. The *zitP^CS^* tetracysteine mutant allele was PCR amplified from the pMT335-P*_zitP_-zitP^CS^* using the 2215shCS+2_SacI and CC2215_E primers. This fragment was digested with *Sac*I/*Eco*RI and cloned into *Sac*I/*Eco*RI-digested pX-*dendra2*N2.

pX-*dendra2*N2-*zitP^GAP^*. The *zitP^GAP^* mutant allele was PCR amplified from the pMT335-*zitP^GAP^* using the 2215sh+2_SacI and CC2215_E primers. This fragment was digested with *Sac*I/*Eco*RI and cloned into *Sac*I/*Eco*RI-digested pX-*dendra2*N2.

pX-*dendra2*N2-*cpaM*. The *cpaM*-coding sequence (nt 3708801-3709982) was PCR amplified using the 3440_+2_SacI and 3440_Eco primers. This fragment was digested with *Sac*I/*Eco*RI and cloned into *Sac*I/*Eco*RI-digested pX-*dendra2*N2.

pMT463*-zitP*. The *zitP*-coding sequence (nt 2444803–2445738,) was PCR amplified from the NA1000 genome using the 2215sh_NdeI and CC2215_E primers. This fragment was digested with *Nde*I/*Eco*RI and cloned into *Nde*I/*Eco*RI-digested pMT463 (Thanbichler et al., 2007).

pMT463*-zitP^1-133^*. The *zitP*-coding sequence (nt 2445340–2445738,) was PCR amplified from the NA1000 genome using the 2215sh_NdeI and 2215_DUF_RI primers. This fragment was digested with *Nde*I/*Eco*RI and cloned into *Nde*I/*Eco*RI-digested pMT463 (Thanbichler et al., 2007).

Synthetic fragments

*2215^CS^:*

Aacatatgtcgatccgcaaggcgcgtcactgggctttctggacatcagggttaaaatcagcgaccccagtaagttatgtggggttcgttcgattcgcggccatgatactgacctccccggagtccgccagccgctatttcgtcgacgactccaaggtcgggccggacggtcgcgtcgtgcgttccgcctcttccggcaatcgctggaccgccttcaaggacgaagctgaagagctgctcgacctcttcgaagagcctgccgccgccagcgccagatcccagggtgatcgcgacgaagccgcggaagaagccgtcgccgccgaggccgaagagccaccggtcagcgcgcttccgggcgaagaacttctt

*2215^CA^:*

aacatatgtcgatccgcaaggcgcgtcactgggctttctggacatcagggttaaaatcagcgaccccagtaagttatgtggggttcgttcgattcgcggccatgatactgaccgccccggaggccgccagccgctatttcgtcgacgactccaaggtcgggccggacggtcgcgtcgtgcgtgccgcctctgccggcaatcgctggaccgccttcaaggacgaagctgaagagctgctcgacctcttcgaagagcctgccgccgccagcgccagatcccagggtgatcgcgacgaagccgcggaagaagccgtcgccgccgaggccgaagagccaccggtcagcgcgcttccgggcgaagaacttctt

**References**

Aldridge, P., R. Paul, P. Goymer, P. Rainey, and U. Jenal. 2003. Role of the GGDEF regulator PleD in polar development of Caulobacter crescentus. *Mol Microbiol*. 47:1695-708.

Biteen, J.S., E.D. Goley, L. Shapiro, and W.E. Moerner. 2012. Three-dimensional super-resolution imaging of the midplane protein FtsZ in live *Caulobacter crescentus* cells using astigmatism. *Chemphyschem*. 13:1007-12.

Bowman, G.R., L.R. Comolli, J. Zhu, M. Eckart, M. Koenig, K.H. Downing, W.E. Moerner, T. Earnest, and L. Shapiro. 2008. A polymeric protein anchors the chromosomal origin/ParB complex at a bacterial cell pole. *Cell*. 134:945-55.

Davis, N.J., Y. Cohen, S. Sanselicio, C. Fumeaux, S. Ozaki, J. Luciano, R.C. Guerrero-Ferreira, E.R. Wright, U. Jenal, and P.H. Viollier. 2013. De- and repolarization mechanism of flagellar morphogenesis during a bacterial cell cycle. *Genes Dev*. 27:2049-62.

Evinger, M., and N. Agabian. 1977. Envelope-associated nucleoid from *Caulobacter crescentus* stalked and swarmer cells. *J Bacteriol*. 132:294-301.

Faulds-Pain, A., C. Birchall, C. Aldridge, W.D. Smith, G. Grimaldi, S. Nakamura, T. Miyata, J. Gray, G. Li, J.X. Tang, K. Namba, T. Minamino, and P.D. Aldridge. 2011. Flagellin redundancy in Caulobacter crescentus and its implications for flagellar filament assembly. *J Bacteriol*. 193:2695-707.

Fioravanti, A., C. Fumeaux, S.S. Mohapatra, C. Bompard, M. Brilli, A. Frandi, V. Castric, V. Villeret, P.H. Viollier, and E.G. Biondi. 2013. DNA binding of the cell cycle transcriptional regulator GcrA depends on N6-adenosine methylation in Caulobacter crescentus and other Alphaproteobacteria. *PLoS Genet*. 9:e1003541.

Fumeaux, C., S.K. Radhakrishnan, S. Ardissone, L. Theraulaz, A. Frandi, D. Martins, J. Nesper, S. Abel, U. Jenal, and P.H. Viollier. 2014. Cell cycle transition from S-phase to G1 in *Caulobacter* is mediated by ancestral virulence regulators. *Nat Commun*. 5:4081.

Gonzalez, D., and J. Collier. 2014. Effects of (p)ppGpp on the progression of the cell cycle of *Caulobacter crescentus*. *J Bacteriol*. 196:2514-25.

Holtzendorff, J., D. Hung, P. Brende, A. Reisenauer, P.H. Viollier, H.H. McAdams, and L. Shapiro. 2004. Oscillating global regulators control the genetic circuit driving a bacterial cell cycle. *Science*. 304:983-7.

Hung, D.Y., and L. Shapiro. 2002. A signal transduction protein cues proteolytic events critical to Caulobacter cell cycle progression. *Proc Natl Acad Sci U S A*. 99:13160-5.

Kirkpatrick, C.L., D. Martins, P. Redder, A. Frandi, J. Mignolet, J.B. Chapalay, M. Chambon, G. Turcatti, and P.H. Viollier. 2016. Growth control switch by a DNA-damage-inducible toxin-antitoxin system in *Caulobacter crescentus*. *Nat Microbiol*. 1:16008.

Lubin, E.A., J.T. Henry, A. Fiebig, S. Crosson, and M.T. Laub. 2015. Identification of the PhoB Regulon and Role of PhoU in the Phosphate Starvation Response of *Caulobacter crescentus*. *J Bacteriol*. 198:187-200.

Mangan, E.K., M. Bartamian, and J.W. Gober. 1995. A mutation that uncouples flagellum assembly from transcription alters the temporal pattern of flagellar gene expression in Caulobacter crescentus. *J Bacteriol*. 177:3176-84.

Murray, S.M., G. Panis, C. Fumeaux, P.H. Viollier, and M. Howard. 2014. Computational and genetic reduction of a cell cycle to its simplest, primordial components. *PLoS Biol*. 11:e1001749.

Radhakrishnan, S.K., M. Thanbichler, and P.H. Viollier. 2008. The dynamic interplay between a cell fate determinant and a lysozyme homolog drives the asymmetric division cycle of Caulobacter crescentus. *Genes Dev*. 22:212-25.

Simon, R., U. Priefer, and A. Puhler. 1983. A Broad Host Range Mobilization System for *In Vivo* Genetic Engineering: Transposon Mutagenesis in Gram Negative Bacteria. *Nat Biotech*. 1:784-791.

Skerker, J.M., and L. Shapiro. 2000a. Identification and cell cycle control of a novel pilus system in *Caulobacter crescentus*. *Embo J*. 19:3223-3234.

Skerker, J.M., and L. Shapiro. 2000b. Identification and cell cycle control of a novel pilus system in *Caulobacter crescentus*. *EMBO J*. 19:3223-34.

Thanbichler, M., A.A. Iniesta, and L. Shapiro. 2007. A comprehensive set of plasmids for vanillate- and xylose-inducible gene expression in *Caulobacter crescentus*. *Nucleic Acids Res*. 35:e137.

Viollier, P.H., and L. Shapiro. 2003. A lytic transglycosylase homologue, PleA, is required for the assembly of pili and the flagellum at the Caulobacter crescentus cell pole. *Mol Microbiol*. 49:331-45.

Viollier, P.H., N. Sternheim, and L. Shapiro. 2002a. A dynamically localized histidine kinase controls the asymmetric distribution of polar pili proteins. *EMBO J*. 21:4420-8.

Viollier, P.H., N. Sternheim, and L. Shapiro. 2002b. Identification of a localization factor for the polar positioning of bacterial structural and regulatory proteins. *Proc Natl Acad Sci U S A*. 99:13831-6.

Viollier, P.H., M. Thanbichler, P.T. McGrath, L. West, M. Meewan, H.H. McAdams, and L. Shapiro. 2004. Rapid and sequential movement of individual chromosomal loci to specific subcellular locations during bacterial DNA replication. *Proc Natl Acad Sci U S A*. 101:9257-62.

Wheeler, R.T., and L. Shapiro. 1999. Differential localization of two histidine kinases controlling bacterial cell differentiation. *Mol Cell*. 4:683-94.
